# Rosai-Dorfman Disease of the Breast

**DOI:** 10.7759/cureus.1153

**Published:** 2017-04-11

**Authors:** Eileen E Delaney, Anne Larkin, Sue MacMaster, Ali Sakhdari, Carolynn M DeBenedectis

**Affiliations:** 1 Department of Radiology, University of Massachusetts Medical School; 2 Department of Surgery, University of Massachusetts Medical School; 3 Department of Pathology, University of Massachusetts Medical School

**Keywords:** rosai-dorfman, sinus histiocytosis with massive lymphadenopathy, breast, inflammatory disorder, non-malignant breast disease

## Abstract

Rosai-Dorfman disease (also known as sinus histiocytosis with massive lymphadenopathy) is a rare benign proliferative disorder of histiocytes that typically involves the lymph nodes and can also involve extranodal sites. Rosai-Dorfman disease confined to the breast is extremely rare, but important to recognize as it can mimic malignancy. We present the case of a 63-year-old woman who presented with a palpable breast lump that was highly suspicious for malignancy based on mammogram and ultrasound appearance. Biopsy revealed inflammatory tissue with lymphoplasmacytic and histiocytic predominance. The diagnosis of Rosai-Dorfman was made based on characteristic staining of histiocytes with S-100 and the presence of emperipolesis. Early recognition of this benign disease entity spared the patient further investigation and surgical intervention.

## Introduction

Rosai-Dorfman disease (also known as sinus histiocytosis with massive lymphadenopathy) is a rare benign proliferative disorder of histiocytes that typically involves the lymph nodes and can also involve extranodal sites. Rosai-Dorfman disease confined to the breast is extremely rare, but important to recognize as it can mimic malignancy. Historically, the diagnosis of Rosai-Dorfman has been made after excisional biopsy. However, a pathologic diagnosis can be made with core biopsy. It is important to diagnose this entity early because patients can be treated conservatively.

## Case presentation

A 63-year-old female presented to her obstetrician/gynecologist (OB/GYN) after feeling a lump in the upper outer quadrant of her right breast. She denied breast pain, skin changes, nipple discharge, and systemic symptoms. There was no family history of breast cancer. On exam, there was a firm, mobile 1.5 cm mass in the upper outer quadrant of the right breast. No additional masses were palpated. Her last mammogram had been two years and four months prior, and all prior mammograms were negative for malignancy. A mammogram and ultrasound were ordered and the patient was referred to a breast surgeon.

### Investigations/imaging findings

**Figure 1:**
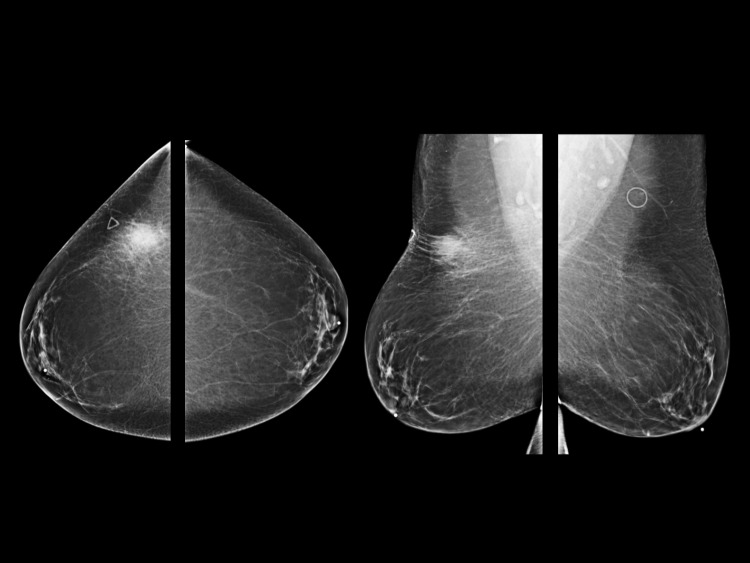
Full field digital diagnostic mammogram demonstrates focal asymmetry in the upper outer quadrant of the right breast corresponding to the palpable marker.

**Figure 2:**
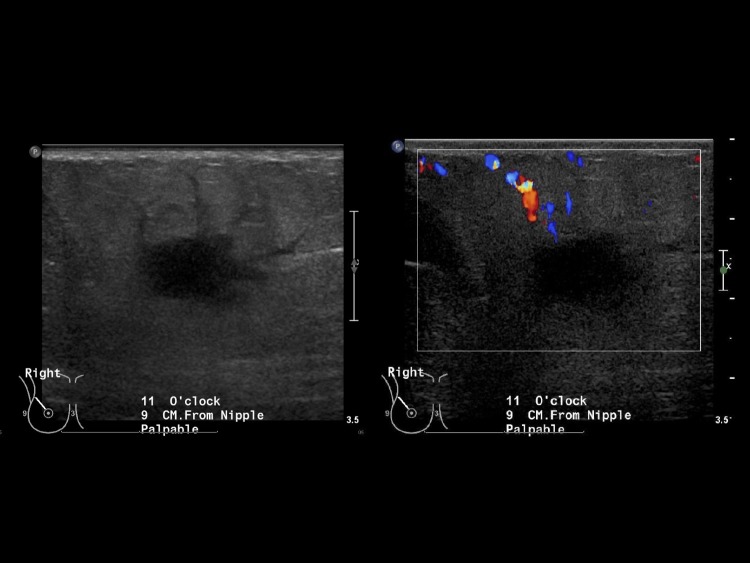
Grayscale and color ultrasound images demonstrate a solid mass with an irregular shape and surrounding edema at site of mammographic and clinically palpable mass, 11 o’clock 9 cm from the nipple.

The patient’s mammogram showed a focal asymmetry in the upper outer quadrant of the right breast corresponding to the palpable marker (Figure 1). Ultrasound of the area demonstrated a solid mass with irregular borders and surrounding edema at 11 o’clock 9 cm from the nipple (Figure 2).

An ultrasound-guided core biopsy was performed. A coil marker was placed in the mass, and a post-biopsy mammogram confirmed that this corresponded with the lesion of interest.

**Figure 3:**
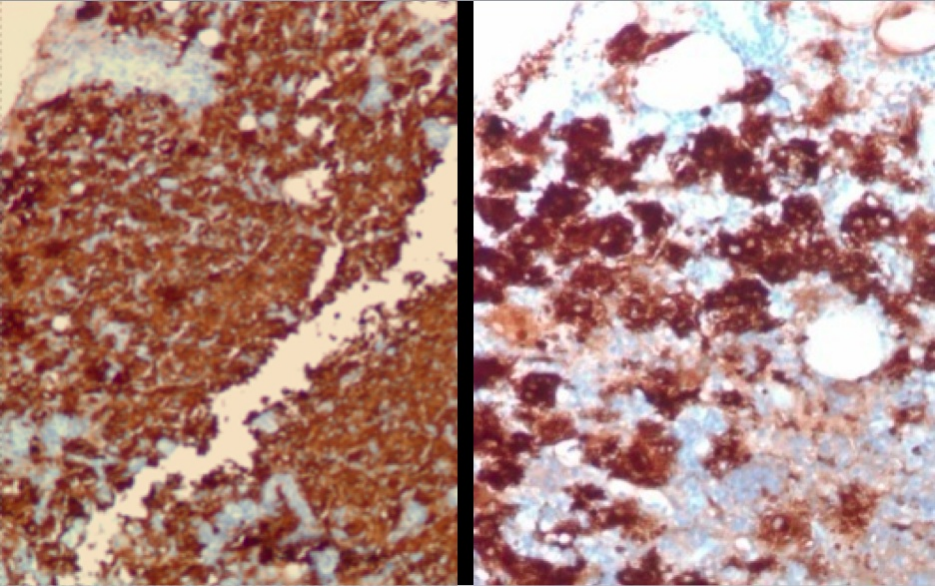
Breast biopsy demonstrating sheets of medium to large histiocytic cells staining diffusely and strongly with S100 protein (100X, 200X).

**Figure 4:**
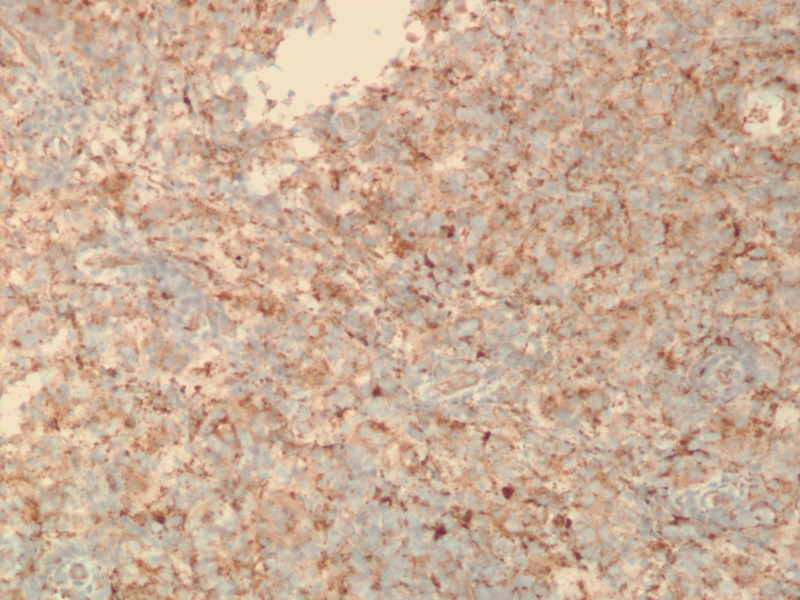
Breast parenchyma with sheets of medium to large histiocytic cells demonstrating faint staining with CD68 (100X).

The core biopsy specimens revealed granulation tissue with extensive acute and chronic inflammation. Specifically, the specimens contained marked lymphoplasmacytic and histiocytic inflammation. There was strong and diffuse staining of histiocytes with S100 (Figure 3), and a subset of the histiocytes stained with CD68 (Figure 4). There was no evidence of malignancy.

The pathology was deemed to be benign and concordant with imaging by the radiologist.

Seven months later, the patient presented to her OB/GYN reporting a one-month history of skin discoloration and induration in the upper inner quadrant of the right breast. Physical exam revealed a 4 × 4 cm area of purple skin discoloration and induration. A separate 2 cm nodule was palpated in the upper outer quadrant of the same breast, corresponding to her known inflammatory lesion. She was again referred to the breast surgeon.

When the patient met with the breast surgeon, the new palpable mass at 12 o’clock in the right breast measured approximately 2.5 cm, and there was an associated maculopapular skin lesion. The previously biopsied lesion in the upper outer quadrant demonstrated apparent growth compared to the exam seven months prior. Given the development of a new mass and apparent growth of the known lesion, imaging and biopsy of both masses were recommended.

**Figure 5:**
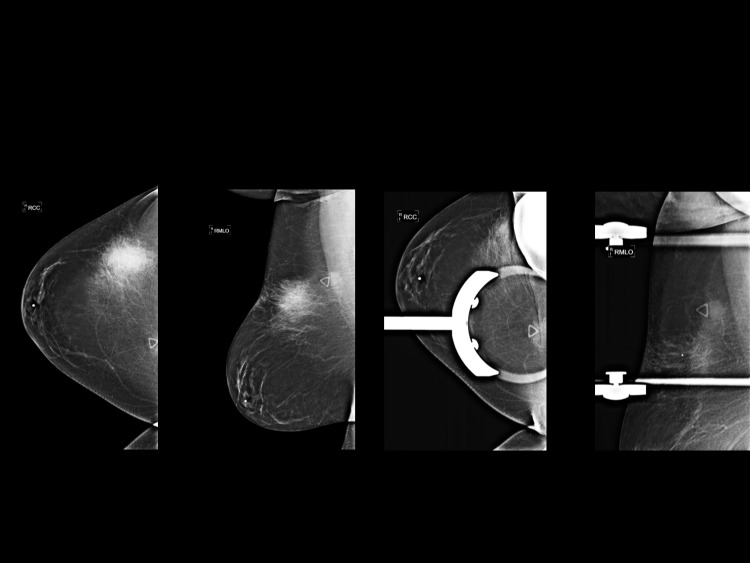
Full field digital diagnostic mammogram and spot compression views demonstrate a focal asymmetry without calcifications.

**Figure 6:**
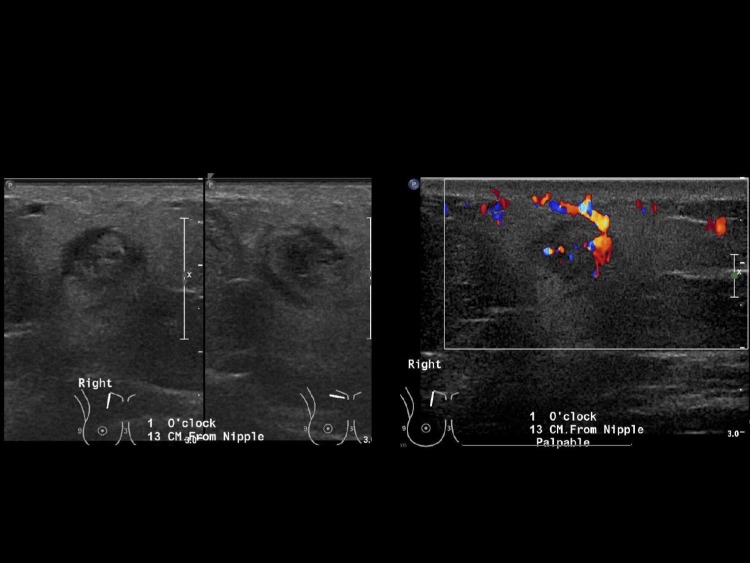
Grayscale and color ultrasound images demonstrate a solid mass with internal vascularity at 1 o’clock 13 cm from the nipple.

The subsequent mammogram showed a focal asymmetry which persisted on spot compression views, corresponding to the new palpable right breast mass (Figure 5). There were no calcifications within the lesion. Ultrasound revealed a solid mass with internal vascularity measuring 10 × 9 × 8 mm at 1 o’clock 13 cm from the nipple (Figure 6). Two overlying skin lesions were also noted.

The previously biopsied mass in the upper outer quadrant demonstrated interval increase in size, but was otherwise unchanged in appearance on mammogram and ultrasound. Thus the decision was made to re-biopsy the 11:00 lesion and biopsy the new 1:00 lesion.

Ultrasound-guided core biopsy was performed on the new mass at 1 o’clock 13 cm from the nipple. A BARD Venus micromarker was placed at the site of biopsy.

The previously biopsied mass at 11 o’clock 9 cm from the nipple was also biopsied. A Senorx ribbon micromarker was placed at the site of biopsy.

A post-biopsy mammogram confirmed appropriate placement of the ribbon micromarker. Due to its posterior location, the Venus micromarker was only visible on the mediolateral oblique (MLO) view. However, it was in a superior and posterior position, which correlates with the expected location in the upper, inner breast.

### Pathology

**Figure 7:**
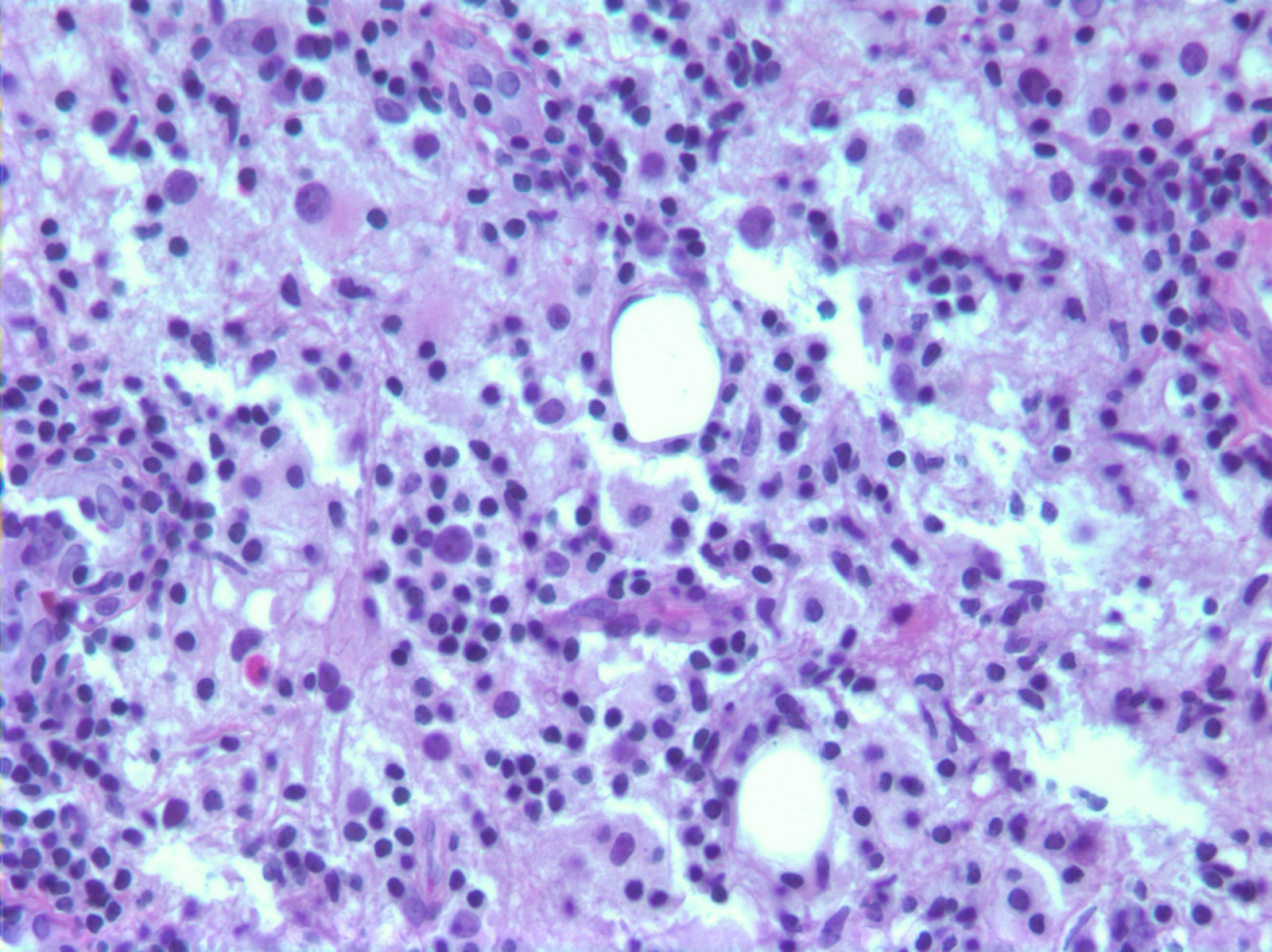
Breast tissue totally replaced by sheets of Rosai-Dorfman histiocytes, small lymphocytes, and scattered plasma cells. Many histiocytes show the emperipolesis of lymphocytes (H&E stain, 400X). H&E - Hemotoxylin and Eosin

Specimens from both lesions demonstrated marked lymphoplasmacytic and histiocytic inflammation. Strong staining of histiocytes with S-100 was again seen. Emperipolesis (phagocytosis of lymphocytes by histiocytes) was also present in the specimens (Figure 7). The final diagnosis for both lesions was extranodal Rosai-Dorfman disease. Carcinoma and lymphoma were ruled out.

### Treatment

Based on these findings, the patient was advised that no surgical intervention was necessary. She followed up with an oncologist who ordered a computed tomography (CT) of the neck, chest, abdomen, and pelvis to evaluate for additional sites of involvement. Two soft tissue nodules were identified in the subcutaneous tissues, one overlying the left anterior superior iliac spine, and the other adjacent to the posterior aspect of the left external oblique muscle. The former was most likely related to recent trauma. No lymphadenopathy or solid organ involvement was identified.

Possible treatment strategies including close observation, empiric trial of steroids, radiation and chemotherapy regimens were discussed at tumor board. The decision was made to opt for close observation. The two subcutaneous nodules identified on the CT of the abdomen and pelvis were not biopsied as recent trauma was thought to be their etiology.

### Outcome and follow-up

At the time of publication, the patient is alive and well. She has not received any therapy for her Rosai-Dorfman disease.

## Discussion

Rosai-Dorfman disease is a rare, benign idiopathic proliferative disorder affecting histiocytes that has been recognized as a distinct entity since 1969 when it was described by Rosai and Dorfman [1]. The most common presentation is painless cervical lymphadenopathy, but extranodal involvement is not uncommon [2]. It has been reported that 23% of cases have exclusively extranodal involvement [3]. The disease typically follows a benign course with spontaneous resolution occurring in most cases. However, some patients may experience progressive disease with solid organ involvement. These cases carry a worse prognosis and may require surgical treatment. In particular, the involvement of the kidneys, lower respiratory tract, and liver are poor prognostic factors [2]. In very rare cases, Rosai-Dorfman disease can be fatal.

Rosai-Dorfman disease limited to the breast is an extremely rare entity. Patients typically present with painless palpable breast masses, although several cases have been discovered in asymptomatic women on screening mammogram [4-5]. Rosai-Dorfman lesions in the breast commonly have an appearance that is indistinguishable from breast carcinoma on mammogram and ultrasound [6-7]. For this reason, most patients have historically undergone excisional biopsy [4]. However, as in this case, a pathologic diagnosis can be made with core biopsy. Characteristic histologic findings include inflammatory infiltrate with strong presence of histiocytes that stain for S-100 protein [8]. The presence of emperipolesis is less commonly seen in extranodal Rosai-Dorfman, but supports the diagnosis when present.

In this case, a CT of the neck, chest, abdomen, and pelvis was obtained for staging purposes and only soft tissue nodules were identified, which were likely hematomas from her recent trauma. This led the tumor board to opt for conservative management. Given that Rosai-Dorfman is a benign disease that often resolves spontaneously, perhaps with early diagnosis more patients in the future will opt for close monitoring as opposed to excision of lesions.

To date, no studies have been performed to determine which imaging modality is best for following known Rosai-Dorfman lesions and detecting disease outside the breast. Some reports indicate that 18F-fluorodeoxyglucose-positron emission tomography/CT is a useful tool for staging, detecting new disease, and following response to treatment [9-10]. In our case, the patient will likely be followed with CT surveillance.

## Conclusions

Rosai-Dorfman disease of the breast is a rare benign inflammatory disorder that can mimic breast cancer clinically and on imaging studies. Strong staining of histiocytes with S100 is characteristic of Rosai-Dorfman. Although nonspecific, emperipolesis is also characteristic and supports the diagnosis when present. Early pathologic diagnosis of Rosai-Dorfman disease of the breast is key because the disease can be treated conservatively.
